# Phytol Suppresses Osteoclast Differentiation and Oxidative Stress through Nrf2/HO-1 Regulation in RANKL-Induced RAW264.7 Cells

**DOI:** 10.3390/cells11223596

**Published:** 2022-11-14

**Authors:** Eun-Nam Kim, Nguyen Minh Trang, Heesun Kang, Ki Hyun Kim, Gil-Saeng Jeong

**Affiliations:** 1 College of Pharmacy, Chungnam National University, Daejeon 34134, Republic of Korea; 2 School of Pharmacy, Sungkyunkwan University, Suwon 16419, Republic of Korea

**Keywords:** phytol, Nrf2, oxidative stress, siRNA, osteoclast

## Abstract

Osteoporosis is a systemic skeletal disorder where osteoclasts are prevalent among osteoblasts. Oxidative stress is one of the main causes of osteoporosis, and nuclear factor erythroid-2-related factor 2 (Nrf2) is the master regulator of antioxidant responses. Phytol, a diterpene isolated from *Stevia rebaudiana* leaves, has many biological effects, including antimicrobial, antioxidant, and anti-inflammatory effects. This study investigated the crosstalk between Nrf2 and osteoclast differentiation in the presence of phytol. Phytol inhibited osteoclast differentiation through TRAP-positive and F-actin formation. The expression of anti-nuclear factor of activated T cells-c1 (NFATc1) and c-Fos was suppressed by phytol, as shown using Western blot and RT-PCR analysis. Phytol inhibited oxidative stress by suppressing reactive oxidant species (ROS) accumulation while recovering antioxidant enzymes, including superoxide dismutase and catalase. Additionally, phytol ameliorated osteoclast-specific differentiation, function, and oxidative stress through Nrf2 regulation by siRNA transfection. In conclusion, these data demonstrate the inhibitory effect of phytol on osteoclast differentiation through Nrf2 regulation, suggesting its potential use in oxidative stress-related osteoporosis and bone diseases.

## 1. Introduction

New bone formation at resorption sites is balanced throughout the lifespan to maintain skeletal mass and strength, where osteoclasts and osteoblasts are involved in bone resorption and bone formation, respectively [1]. Once the equilibrium is disturbed, osteoclasts prevail over osteoblasts, leading to osteoporosis [2]. Osteoclast differentiation is triggered by the binding of receptor activator of nuclear factor kappa-B ligand (RANKL) to its receptor on osteoclastic progenitors, which is caused by the chronic gradual deterioration of the functional capacity related to oxidative stress [3,4]. Osteoporosis activates many downstream signaling pathways, including reactive oxidant species (ROS), an intracellular signaling molecule that regulates osteoclast differentiation, and important secondary messengers that cause oxidative damage to proteins and DNA [5,6]. Nuclear factor erythroid-2-related factor 2 (Nrf2) reduces intracellular ROS, diminishes RANKL-induced osteoclastogenesis, and decreases osteoclastic gene expression via antioxidant response amelioration [6].

Cells have many protective mechanisms against oxidative stressors, including the induction of cytoprotective enzymes, such as antioxidative enzymes, through cytoplasmic oxidative stress system activation [7]. Nrf2 transcriptionally controls the expression of many cytoprotective enzymes, such as heme oxygenase-1 (HO-1) [7]. Nrf2 is ubiquitously expressed in almost all cell types, including osteoblasts, osteocytes, and osteoclasts [8]. Regulating intracellular ROS signaling activates Nrf2/Kelch-like ECH-associated protein (Keap1) signaling pathway during osteoclast differentiation [9]. Activated Nrf2 translocates into the nucleus and activates the transcription of many genes, including antioxidant and detoxification enzymes, whose promoters contain an antioxidant response element [10,11]. RANKL stimulation attenuates Nrf2 gene expression to favor ROS signaling [9]. In the normal bone state, the cells constantly fight against excess ROS via intracellular antioxidant enzymes, such as superoxide dismutase (SOD) and catalase (CAT), and the balance of the intracellular levels of these enzymes is necessary for normal conditions [12]. Nrf2 deletion increases bone resorption, RANKL expression, and osteoclast count; reduces the mechanical properties of the skeletal system and load-driven bone formation; and impairs fracture healing in mice [13,14,15,16]. In addition, Nrf2 deletion in osteoclast precursor cells derived from Nrf2-knockdown mice induces oxidative stress and RANKL-induced osteoclast differentiation [17]. Thus, Nrf2 plays an important role in bone homeostasis regulation and oxidative stress reduction in bone cells.

To discover pharmacologically active natural products from diverse natural resources [18,19,20,21], we focused on *Stevia rebaudiana* Bertoni, a branched bushy shrub belonging to the Asteraceae family. Steviol, a diterpenoid glycoside (stevioside) isolated from *S. rebaudiana* leaves, is sweeter than sucrose with zero calories and is used as a sweetening agent, particularly for diabetic and obese people with a strict diet [22]. In this study, phytochemical investigation of *S. rebaudiana* leaves reported the isolation of a diterpenoid, phytol. Phytol has many biomedical effects such as antioxidant, anxiolytic, anticonvulsant, antimicrobial, antinociceptive, and anti-inflammatory effects [23]. However, the effect of phytol on osteoclast differentiation and bone formation has rarely been investigated.

Currently, most osteoporosis drugs block bone resorption by reducing the formation or activity of osteoclasts, and bisphosphonate drugs are typically used [24]. Although bisphosphonate drugs are used as bone resorption inhibitors through their direct apoptosis effect on osteoclasts, they have to be taken for a long period of time and have high bone binding strength, which causes gastrointestinal problems such as esophageal inflammation and ulcers along with jaw bone necrosis even after the patient stops the drug [25]. Therefore, it is urgent to develop a new treatment that can replace the current treatment.

Therefore, to investigate this hypothesis, we investigate the role of phytol in RANKL-induced osteoclasts in RAW264.7 cells in vitro and propose the possibility of phytol as a relatively safe natural product derived therapeutic agent that can replace conventional therapeutics.

## 2. Materials and Methods

### 2.1. Reagents and Chemicals

Recombinant soluble RANKL ligand (sRANKL) was acquired from PeproTech EC, Ltd. (PeproTech EC, Ltd.; London, UK). The acid phosphatase assay kit, 3-(4,5-Dimethylthiazol-2-yl)-2,5-diphenyltetrazoliumbromide (MTT), leukocyte acid phosphatase, 4-6-diamidino-2-phenylindole (DAPI), ascorbic acid, and β-glycerophosphate were purchased from Sigma-Aldrich Fine Chemicals (Sigma-Aldrich Fine Chemicals; Saint Louis, MO, USA). Dulbecco’s modified Eagle’s medium (DMEM), minimum essential medium alpha (α-MEM), and fetal bovine serum (FBS) were purchased from Welgene Biosciences (Welgene Biosciences; Daegu, Korea). TRIZOL reagent was purchased from Welgene Biosciences (Welgene Biosciences; Daegu, Korea) and TOPscript™ RT DryMIX (dT18 plus) was purchased from Enzynomics (Enzynomics; Daejeon, Korea). TB Green^®^ Premix Ex Taq™ II (Tli RNase H Plus) was purchased from Takara (Takara, Tokyo, Japan). Osteo Assay Plate was purchased from Corning Inc. (Corning Inc; Corning, NY, USA). Acid phosphatase 5, tartrate-resistant (*acp5*), ATPase H+ transporting V0 subunit D2 (*atp6d0v2*), and dendritic cell-specific transmembrane protein (*dcstamp*) were purchased from Biomedic Co. (Biomedic Co; Bucheon, Korea). Primary antibodies against Nrf2, HO-1, and β-actin were purchased from Santa Cruz Biotechnology, Inc. (Santa Cruz Biotechnology, Inc; Santa Cruz, CA, USA), while those against CAT, SOD, anti-nuclear factor of activated T cells-c1 (NFATc1), and c-Fos were acquired from BD Biosciences (BD Biosciences; San Jose, CA, USA). An enhanced chemiluminescence (ECL) Western blotting detection system was purchased from Advansta Inc. (Advansta Inc; San Jose, CA, USA).

### 2.2. Plant Materials

*S. rebaudiana* specimens were collected in August 2018 from Namyangju-si (Gyeonggi-do, Korea) and verified by one of the authors (K. H. Kim). A voucher specimen, STBA-08-2018, was deposited in the herbarium of the School of Pharmacy affiliated with Sungkyunkwan University, Suwon, Korea.

### 2.3. Phytol Isolation and Identification

**Figure 1:**
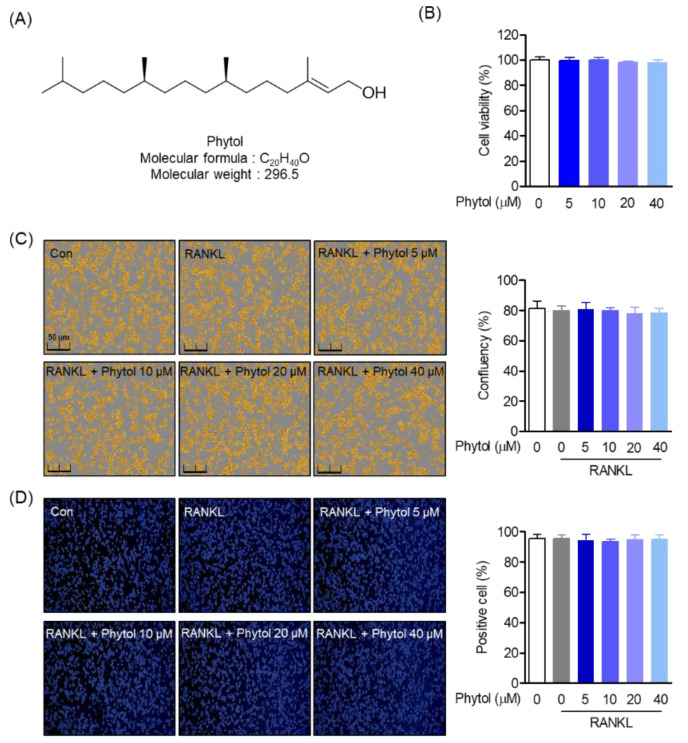
Cell viability and phytol confluency. (**A**) Chemical structure of phytol. (**B**) RAW264.7 viability and (**C**) confluency after 5–40 µM phytol treatment. (**D**) Phytol-positive cell measurement through trypan blue staining analysis. Data are presented as the mean of three independent experiments (Mean ± SD).

Dried *S. rebaudiana* leaves (5 kg) were extracted with 3.0 L 80% aqueous EtOH for 3 days at room temperature, and the suspension was filtered. The filtrate was combined and concentrated under reduced pressure using a rotavapor to obtain the EtOH extract (169.4 g), which was suspended in distilled water (700 mL) and successively solvent-partitioned with hexane, dichloromethane (CH_2_Cl_2_), ethyl acetate (EtOAc), and *n*-butanol (BuOH). Four layers with increasing polarity were obtained: hexane-soluble (15.8 g), CH_2_Cl_2_-soluble (3.0 g), EtOAc-soluble (8.9 g), and *n*-BuOH-soluble (12.4 g). Phytol was isolated from the *n*-hexane-soluble fraction (15.7 g) using silica gel column chromatography [eluted with a gradient solvent system of hexane/EtOAc (50:1→1:1) and 100% MeOH] to obtain 26 fractions (A–W). Fraction M (173.1 mg) was subjected to reverse-phase C_18_ column chromatography [eluted with a gradient solvent system of MeOH/H_2_O (70%→80%→90%→100% MeOH)] to obtain six subfractions (M1–M6). Phytol (2.0 mg, *t*_R_ = 82.0 min) was obtained from subfraction M6 (16.5 mg) by purification using semi-preparative HPLC (78% MeCN; flow rate: 2 mL/min) with a Phenomenex Luna Phenyl-hexyl 100 Å column (250 × 10 mm^2^, 5 μm; Phenomenex; Torrance, CA, USA). For structural elucidation, nuclear magnetic resonance (NMR) spectra were recorded using a Bruker AVANCE III HD 850 NMR spectrometer (Bruker; Karlsruhe, Germany) with a 5 mm TCI CryoProbe. LC/MS analysis was carried out using an Agilent 1200 Series HPLC system equipped with a diode array detector and a 6130 Series ESI mass spectrometer. The column for LC/MS was an analytical Kinetex C_18_ 100 Å column (100 × 2.1 mm^2^, 5 μm; Phenomenex) with a 0.3 mL/min flow rate. Phytol structure (Figure 1A) was determined by comparing its NMR data with those previously reported [26] and MS data from LC/MS analysis.

#### Phytol

Viscous oil; (+)-ESIMS *m*/*z*: 297.3 [M+H]^+^; ^1^H NMR (850 MHz, CDCl_3_): δ_H_ 0.82 (3H, d, *J* = 6.5 Hz, H-19), 0.83 (3H, d, *J* = 6.5 Hz, H-18), 0.84 (3H, d, *J* = 6.5 Hz, H-17), 0.84 (3H, d, *J* = 6.5 Hz, H-16), 1.03–1.40 (18H, m, H-5–H-14), 1.50 (1H, m, H-15), 1.65 (3H, s, H-20), 1.97 (2H, m, H-4), 4.14 (2H, d, *J* = 6.5 Hz, H-1), 5.39 (1H, t, *J* = 6.5 Hz, H-2); ^13^C NMR (212.5 MHz; CDCl_3_): δ_C_ 16.1 (C-20), 19.6 (C-18), 19.7 (C-19), 22.6 (C-17), 22.6 (C-16), 24.4–25.1 (C-5, C-9, C-13), 27.9 (C-15), 32.6 (C-7), 32.7 (C-11), 36.6 (C-6), 37.2–37.4 (C-8, C-10, C-12), 39.3 (C-14), 39.8 (C-4), 59.3 (C-1), 123.1 (C-2), 140.2 (C-3).

### 2.4. RAW 264.7 Cell Culture and Osteoclast Differentiation

RAW 264.7 murine macrophage cells were acquired from the American Type Culture Collection (ATCC; Rockville, MD, USA) and seeded at 5 × 10^3^ cells/well in DMEM high glucose supplemented with 10% FBS, 100 U/mL penicillin, and streptomycin. For osteoclast differentiation, the cells were incubated for 24 h at 37 °C in a humidified atmosphere containing 5% CO_2_ before changing to minimum essential medium alpha (α-MEM) supplemented with 10% FBS, 100 U/mL penicillin, and streptomycin. The cells were then treated with 50 ng/mL RANKL and 5, 10, 20, or 40 µM phytol for 5 days. The medium was refreshed every day. TRAP staining, activity analysis, and other experimental methods were used to identify osteoclast differentiation.

### 2.5. Cell Viability and Confluency

The cytotoxic effect of phytol on RAW 264.7 cell was determined using the 4,5-dimethylthiazol-2-thiazolyl-5-diphenyltetrazolium bromide (MTT) assay and cell confluency using Incucyte^®^ Live-Cell analysis systems (Satorius; Göttingen, Germany) before further experiments. Briefly, in MTT assay, 5 × 10^3^ cells/well were seeded in 96-well plates and maintained at 37 °C in a humidified 5% CO_2_ incubator for 24 h and then treated with or without 5–40 µM phytol in the presence or absence of 50 ng/mL RANKL for 5 days. MTT (5 mg/mL) was then added to each well and incubated for 4 h. The supernatant was replaced with dimethyl sulfoxide (DMSO) to dissolve the formazan crystals. Cell viability was measured at 540 nm using a microplate reader (TECAN infinity pro2000; Männedorf, Switzerland). The data are represented in triplicate experiments. For trypan blue viability assay and cell count assays, the cells were phytol-treated and incubated for 5 days, followed by the addition of 0.4% trypan blue solution to 1% volume of the wells. The cells were detached from the wells by serial trituration, and 10 μL cell suspension was loaded on a hemocytometer to calculate the percentage of viable (unstained), dead (stained), and total cells.

### 2.6. Immunofluorescence Analysis

RAW264.6 cells were fixed in 4% paraformaldehyde for 20 min and incubated in Triton-X100 for 10 min to penetrate the cell membrane, after treatment with the indicated concentration of phytol, and then goat serum blocking solution was added to each well for 1 h. Next, cells were incubated with rabbit anti-Nrf2 antibody (Abcam; Cambridge, MA, USA; 1:100) overnight at 4 °C and fluorescein isothiocyanate (FITC)-conjugated anti-rabbit IgG (Abcam; Cambridge, MA, USA; 1:1000) images were captured with a fluorescence microscope (Olympus; Tokyo, Japan) for 1 h at room temperature in the dark.

### 2.7. Cytosolic and Nuclear Protein Extraction

RAW264.7 cells were seeded at 1 × 10^6^ cells/mL in 6-well plates. Then, the collected cells were lysed on ice for 20 min using RIPA (radioimmunoprecipitation assay) buffer (Thermo Fisher Scientific, Waltham, MA, USA) and the isolated cytoplasm and nuclei were treated with the NE-PER nuclear and cytoplasmic extraction reagent kit (Pierce Biotechnology; Rockford, IL, USA) was used according to the manufacturer’s instructions.

### 2.8. Cell Migration

RAW 264.7 cells (1 × 10^3^ cells/well) were seeded in 6-well plates and incubated for 24 h, followed by verticality scratching. Subsequently, they were treated with 50 ng/mL RANKL with or without phytol at the experimental concentration. The number of migrating cells is marked in yellow on the red line. Cell migration was measured on the first and fifth days after phytol treatment using an Incucyte^®^ Live-Cell analysis system. The only RANKL-treated group was used as a positive control. In addition, the migration of RAW264.7 cells was analyzed using a migration transwell migration assay kit (Corning; Corning, NY, USA). After incubation at 37 °C for 18 h, samples and RANKL were treated in the same manner and measured on the first and fifth days after phytol treatment using an Incucyte^®^ Live-Cell assay system.

### 2.9. TRAP Staining and Activity

RAW 264.7 cells were cultured in 24-well culture plates at 1 × 10^3^ cells/well in DMEM supplemented with 10% FBS, 100 U/mL penicillin, and streptomycin. After incubation for 24 h, the cells were transferred to differentiated medium containing 50 ng/mL RANKL and processed or unprocessed with 5–40 µM phytol for 5 days. The medium was refreshed daily. Then, the medium was removed, and the cells were washed twice with phosphate-buffered saline (PBS) before being fixed with 4% formaldehyde for 15 min and washed once with PBS. After incubation for 24 h, the cells were transferred to osteoclast differentiation medium and treated with different phytol concentrations for 5 days. Then, the cells were washed twice with PBS before being fixed with 4% formaldehyde for 15 min. Osteoclast differentiation was measured using an Acid Phosphatase Assay kit (Cat. No. CS 0740, Sigma-Aldrich Fine Chemicals (Saint Louis, MO, USA) and Acid Phosphatase Leukocyte (Cat. No. 387A-1KT, Sigma-Aldrich Fine Chemicals (Saint Louis, MO, USA) according to the manufacturer’s instructions.

### 2.10. Actin Ring and DAPI Staining

Alexa 488 phalloidin (Invitrogen; Carlsbad, CA, USA) was used to stain the ring part of osteoclasts. Briefly, the cells were maintained in a medium containing processed or unprocessed phytol for 5 days and fixed in 4% formaldehyde for 15 min before being permeabilization with 0.5% Triton X-100 solution. The experiment was carried out in dark by staining the cells with Alexa 488 phalloidin for 1 h, followed by contrast staining of nuclei with DAPI for 30 min before washing with cold PBS. A fluorescence microscope (Nikon Co.; Tokyo, Japan) was used to visualize actin rings and DAPI staining of mature osteoclasts.

### 2.11. Osteoclastic Resorption

RAW 264.7 cells (1 × 10^3^ cells/well) were cultured on a Corning osteo assay plate (Corning, NY, USA). After 24 h of incubation, cells were transferred to α-MEM medium supplemented with 5% FBS and treated with or without the indicated phytol doses and 50 ng/mL RANKL for 7 days. The medium was changed daily. To quantify resorption, the medium was removed, the cells were washed twice with PBS, followed by detachment with 5% sodium hypochlorite for 5 min, washed with PBS, and dried. The resorption area was visualized using a microscope (Olympus; Tokyo, Japan) and measured using the ImageJ software.

### 2.12. Intracellular ROS Measurement

RAW 264.7 (5 × 10^3^ cells/well) were seeded in 24-well plates and incubated for 24 h. Then, cells were treated with RANKL (50 ng/mL) and/or phytol at different concentrations for 1 day in α-MEM supplemented with 10% FBS, 100 U/mL penicillin, and streptomycin. To assess ROS production, 2′, 7′-dichlorodihydrofluorescein diacetate (DCF-DA) (Sigma-Aldrich Fine Chemicals; Saint Louis, MO, USA) was used. The experiments were conducted in dark, and the cells were incubated for 20 min at 37 °C, washed with PBS, and fixed with 4% paraformaldehyde (pH 7.4) for 20 min. The Incucyte^®^ live-cell analysis system was used to measure ROS levels.

### 2.13. Real-Time Quantitative PCR

The expression levels of *dcstamp*, *acp5*, *atp6d0v2*, *ctsk*, *sod*, *and cat* were detected using RT-PCR. TRIzol reagent was used to extract total RNA from cell lysates. NanoDrop (Thermo Scientific; Waltham, MA, USA) and TOPscript™ RT DryMIX (dT18 plus) were used to determine the concentration of mRNA and synthesized cDNA, respectively. A LightCycler 480 (Roche; Basel, Switzerland) was used to perform RT-PCR with TB Green^®^ Premix Ex Taq™ II (Tli RNaseH Plus). The equation for evaluating gene expression was as follows: 2−ΔΔCT, where ΔΔCT = (CT*target* − CT*gapdh*) at time x − (CT*target* − CT*gapdh*) at time 0, where time x represents any time point, and time 0 represents 1 × expression of the gene in the untreated group normalized to that of *gapdh*, a housekeeping gene. The experiment was independently replicated three times. The primers used are listed in Table 1.

### 2.14. Western Blot Analysis

Treated and untreated cells were harvested and lysed in RIPA buffer containing protease inhibitors for 30 min. A nuclear/cytosol fraction kit (Pierce Biotechnology; Rockford, IL, USA) was used to separate the nuclear and cytoplasmic proteins according to the manufacturer’s protocol. The Bradford method was used to measure protein concentration according to the manufacturer’s instructions. Equal amounts of protein from each sample were electrophoresed on 12% sodium dodecyl sulfate polyacrylamide gel and transferred on PVDF membrane (Bio-Rad; Hercules, CA, USA). The membrane was blocked with 5% skim milk and incubating with primary antibodies overnight. Primary antibodies against Nrf2, HO-1, SOD, CAT, NFATc1, and c-Fos were used to detect the expression of each protein, and β-actin was used as a housekeeping gene. The bands were visualized with ECL Western blotting detection reagents (Thermo Fisher Scientific; Waltham, MA, USA) using ImageQuant LAS 4000 (GE Healthcare; Chicago, IL, USA). ImageJ software was used to quantify images of Western blot bands, which were normalized to that of the control.

### 2.15. siRNA Interference

RAW264.7 cells (5 × 10^3^ cells/well) were dispensed in a 6-well plate and incubated for 24 h at 37 °C with 5% CO_2_. siRNA against Nrf2 was transfected using Lipofectamine 2000 (Invitrogen; Carlsbad, CA, USA). Transfection with Nrf2 siRNA (Santa Cruz, TX, USA) was carried out according to Lipofectamine 2000 manufacturer’s instructions. After re-incubation for 6 h in a humidified environment with 5% CO_2_, the cells were harvested for further analysis. Western blotting was used to determine the protein expression.

### 2.16. Statistical Analysis

Statistical analysis was performed by one-way ANOVA using SPSS Statistics 19.0 software (Armonk, NY, USA). Each experiment was repeated three times and expressed as the mean and standard deviation, *p* < 0.05 was considered to indicate statistical significance.

## 3. Results

### 3.1. Cell Viability and Phytol Confluency

To assess the cytotoxic effect of phytol (Figure 1A) on RAW264.7 cells, cell viability and confluency were first investigated. After treatment with 5–40 µM phytol, the MTT and Incucyte^®^ Live-Cell analysis systems were used to test cell viability and confluency, respectively (Figure 1B,C). Cell viability was measured by MTT assay and cell confluency was imaged 24 h after treatment with phytol for 5 days. The positive cell count within the indicated concentrations was determined by trypan blue staining (Figure 1D). The data demonstrated that the indicated phytol concentrations did not exert toxicity to RAW264.7.

### 3.2. Phytol Induces HO-1 Expression by Nrf2 Translocation Promotion

**Figure 2:**
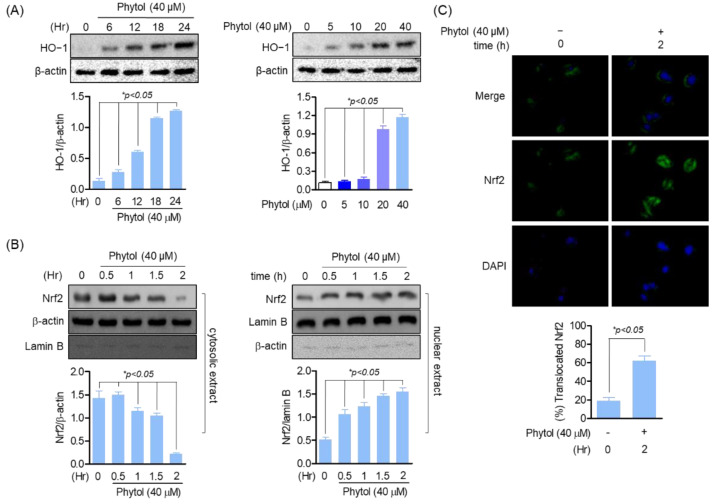
Phytol induces HO−1 expression by Nrf2 translocation promotion. (**A**) Cells (1 × 10^5^ cells/well) were seeded and incubated for 24 h followed by phytol treatment at the indicated concentration for Western blot detection. (**B**) Nuclear and cytoplasmic proteins were extracted according to the instruction of manufacturer (Thermo scientific, Waltham, MA, USA) before loading on sodium dodecyl sulfate polyacrylamide gels to assess the protein expression level. (**C**) After treatment with the indicated 40 μM concentration of phytol within 2 h, Nrf2 immunocytochemistry staining was performed to confirm translocation. Data are presented as the mean of three independent experiments (Mean ± SD).

To investigate the relationship between phytol and antioxidants, we targeted HO-1, a well-known antioxidant. Phytol induced HO-1 expression time- and concentration-dependently (Figure 2A). In particular, HO-1 expression significantly increased in 20 and 40 µM phytol-treated cells. Thus, phytol has a potential antioxidant activity. As HO-1 is a cytoprotective regulator of Nrf2 transcription factors, the relationship between phytol and Nrf2 was investigated. The cytosolic and nuclear extracts of phytol-treated RAW264.7 cells were detected by Western blotting, which indicated that Nrf2 expression decreased in the cytosolic extract of 40 µM phytol-treated cells, whereas it increased in the nuclear extract. Phytol did not affect the expression of the housekeeping genes, β-actin and lamin B (Figure 2B). For more clear evidence of the Nrf2 translocation induction result of phytol, the Nrf2 translocation induction effect of phytol was confirmed through immunocytochemistry staining, these results suggest that the induction of HO-1 expression by phytol is through Nrf2 translocation promotion (Figure 2C).

### 3.3. Inhibitory Effect of Phytol on RANKL-Induced Osteoclast Differentiation and Formation

**Figure 3:**
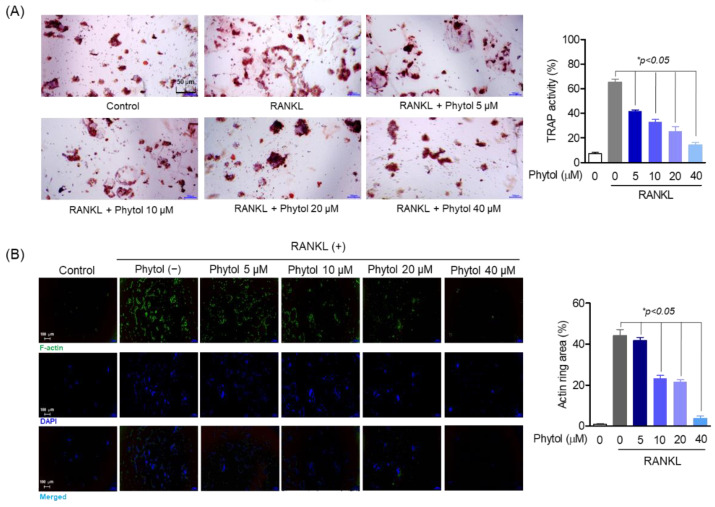
Inhibitory effect of phytol on receptor activator of nuclear factor kappa-B ligand (RANKL)-induced osteoclast differentiation and formation. (**A**) The cells (1 × 10^3^ cells/well) were seeded in 24-well plates and incubated for 24 h. After treatment with phytol at different concentration with or without 50 ng/mL RANKL for 5 days, TRAP staining and activity was carried out. (**B**) To investigate osteoclast formation, the cells were stimulated with RANKL and treated with phytol for 5 days. Alexa 488-Phalloidin (green) and DAPI (blue) was used to stain the actin cytoskeleton and counterstain the nuclei, respectively. Data are presented as the mean of three independent experiments (Mean ± SD). * *p* < 0.05, compared to the RANKL-induced group.

To assess osteoclast differentiation and formation, multinucleated cells were detected by TRAP-positive staining and F-actin formation. The number of TRAP-positive cells decreased phytol concentration-dependently (Figure 3A). Moreover, the phytol-treated cells suppressed F-actin ring formation, while RANKL promoted actin area formation (Figure 3B).

### 3.4. Inhibitory Effect of Phytol on RANKL-Induced Osteoclast Function

**Figure 4:**
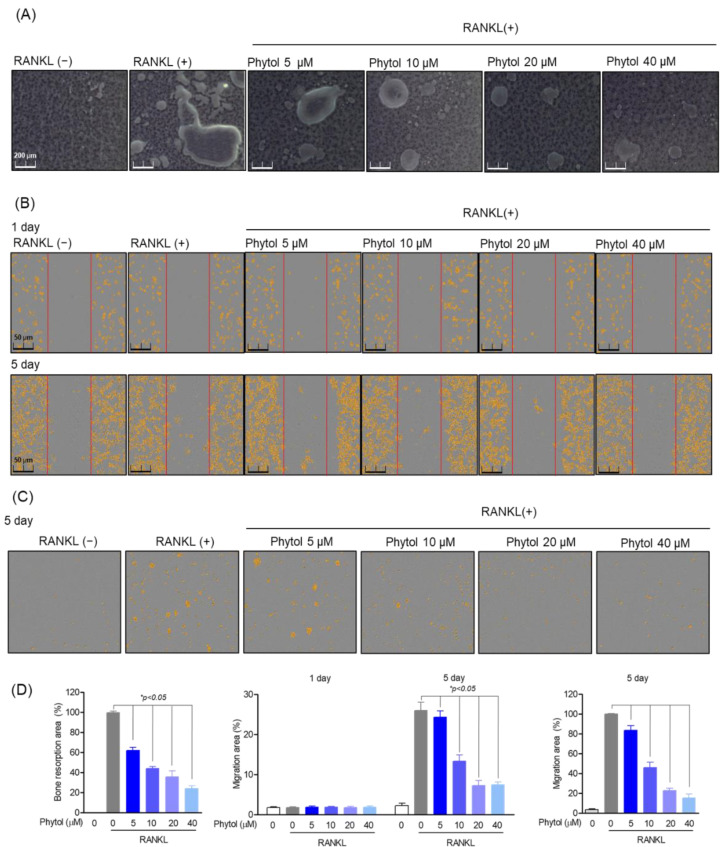
Inhibitory effect of phytol on receptor activator of nuclear factor kappa−B ligand (RANKL)-induced osteoclast function. (**A**) Bone resorption area after treating 5 × 10^3^ cells/well with phytol at the indicated concentration for 5 days. A microscope (Olympus; Tokyo, Japan) and ImageJ program were used to measure the resorption area. (**B**) Incucyte^®^ Live-Cell analysis system was used to measure cell migration on day 1 and day 5 after treating cells in RANKL induced osteoclast model with phytol. (**C**) RANKL-induced osteoclast migration measurement of phytol in transwell. (**D**) Area of bone resorption and osteoclast migration. Data are presented as the mean of three independent experiments (Mean ± SD). * *p* < 0.05 compared to the RANKL-induced group.

To determine whether phytol inhibits osteoclast function, bone resorption and RAW264.7 cell migration were studied. The percentage of bone resorption in the phytol-treated group was significantly lower than that in the RANKL-induced osteoclast differentiation group (positive-control), and this trend was phytol concentration dependent (Figure 4A). Similarly, osteoclast migration, a specific osteoclast function, was studied after 5 days of treatment with phytol at different concentrations in the presence or absence of RANKL. Cell migration decreased compared to that in the only RANKL-treated group (Figure 4B,C), and phytol significantly decreased the bone resorption area and osteoclast movement quantitatively (Figure 4D). These data suggest that phytol suppresses osteoclast function.

### 3.5. Phytol Reduces the Expression of Osteoclast Marker Genes and Transcription Factors

**Figure 5:**
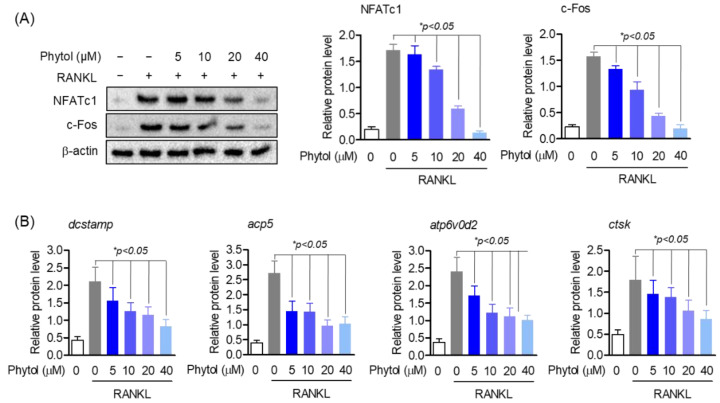
Phytol decreases the expression of osteoclast marker genes and transcript factors. The cells were pretreated with 5−40 µM phytol before stimulating with 50 ng/mL receptor activator of nuclear factor kappa-B ligand (RANKL). (**A**) Western blotting was used to investigate the expression of NFATc1 and c−Fos protein level. (**B**) To detect the mRNA expression of *dcstamp*, *acp5*, *atp6v0d2*, and *ctsk*, RT-PCR was used. Data are presented as the mean of three independent experiments (Mean ± SD).

RANKL activates several transcription factors involved in osteoclast differentiation. Consequently, c-Fos activates the master regulator of osteoclast differentiation, NFATc1. In this study, we investigated the expression of osteoclast marker genes and transcription factors to determine the effect of phytol on osteoclast differentiation. NFATc1 and c-Fos protein levels were detected using Western blot analysis; compared to RANKL-induced differentiated osteoclasts, cells treated with phytol showed reduced expression of these transcription factors (Figure 5A). Moreover, phytol did not affect the expression of the housekeeping gene, β-actin. Expression of osteoclast marker genes, such as dendritic cell-specific transmembrane protein (*dcstamp*), acid phosphatase 5 (*acp5*), ATPase H+ transporting V0 subunit D2 (*atp6d0v2*), and cathepsin K (*ctsk*), was determined using RT-PCR. RANKL stimulation increased *dcstamp*, *acp5*, *atp6d0v2*, and *ctsk* mRNA levels, whereas phytol reduced their expression (Figure 5B).

### 3.6. Reduced Effect of Phytol on Oxidative Stress Markers in RANKL-Induced Osteoclasts

**Figure 6:**
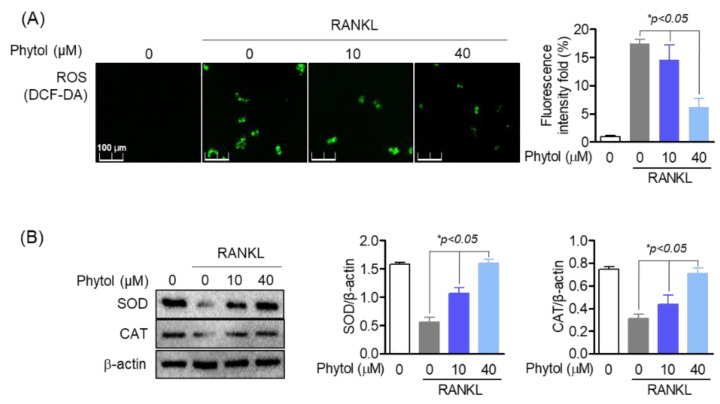
Reduced effect of phytol on oxidative stress markers in receptor activator of nuclear factor kappa−B ligand (RANKL)-induced osteoclasts. (**A**) 2′,7′−dichlorodihydrofluorescein diacetate (DCF-DA) was used to investigate reactive oxygen species (ROS) accumulation in 10−40 µM phytol-treated cells. (**B**) The recovery of antioxidant enzymes including superoxide dismutase (SOD) and catalase (CAT) was measured by Western blot analysis and normalized to that of β-actin. Data are presented as the mean of three independent experiments (Mean ± SD).

ROS function as potent multifunctional signaling molecules in intercellular pathways that regulate osteoclast differentiation. Thus, ROS production was investigated. DCF-DA staining (green) was used to detect ROS accumulation (Figure 6A). The results showed that phytol reduced ROS accumulation concentration-dependently. SOD and CAT are antioxidant enzymes. Western blot analysis revealed that only RANKL treatment reduced SOD and CAT expression (Figure 6B). In the presence of 40 µM phytol, the expression of these antioxidant enzymes was recovered.

### 3.7. Phytol-Mediated Inhibition of Osteoclast Differentiation and Function Is Regulated through Nrf2

**Figure 7:**
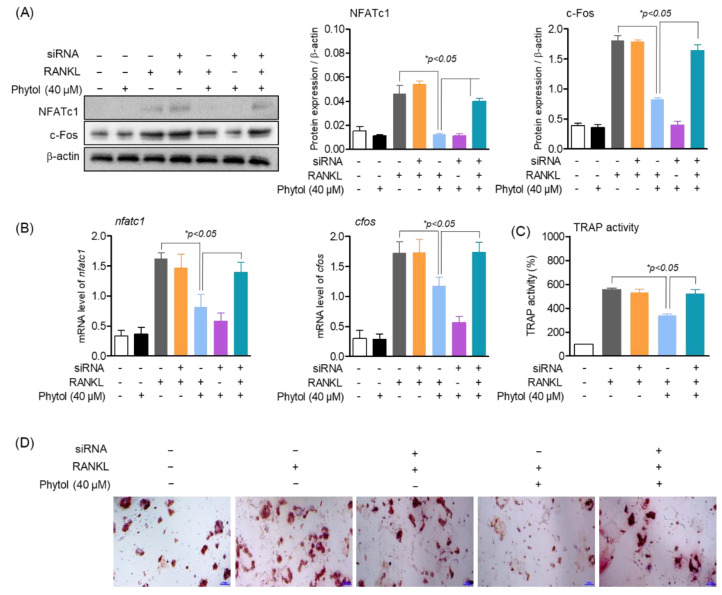
Inhibition of osteoclast differentiation and function by phytol is regulated through Nrf2. (**A**) NFATc1 and c−Fos protein expression were measured by Western blot analysis and normalized to that of β-actin. (**B**) *nfatc1* and *cfos* mRNA expression were detected by RT−PCR. (**C**,**D**) Cells (1 × 10^3^ cells/well) were treated with phytol in the presence of RANKL with or without Nrf2 silencing using siRNA. After 5 days incubation, the cells were assessed for multinucleated osteoclast differentiation using TRAP activity and stained. Data are presented as the mean of three independent experiments (Mean ± SD).

Nrf2 plays an important role in osteoclast differentiation. Activated Nrf2 translocates to the nucleus and activates the transcription of several genes. In this study, we investigated the effect of phytol on osteoclast differentiation and function by regulating Nrf2, NFATc1, and c-Fos, which regulate osteoclast differentiation. The protein expression and mRNA levels of RANKL-induced osteoclasts with or without RANKL increased (Figure 7A,B). However, in RANKL-induced osteoclasts treated with phytol and Nrf2 siRNA, NFATc1 mRNA level and NFATc1 and c-Fos protein expression were decreased, while c-Fos mRNA level remained unchanged. These data suggest that phytol inhibits osteoclast differentiation and function through Nrf2 regulation. Additionally, the effect of phytol on osteoclast differentiation through Nrf2 regulation was investigated by measuring TRAP-positive multinucleated cells and osteoclastic resorption. When Nrf2 was silenced, TRAP activity and bone resorption area were significantly increased compared to those after phytol treatment in the presence of Nrf2. Thus, phytol inhibited osteoclast differentiation and function through Nrf2 regulation (Figure 7C,D).

### 3.8. Phytol-Mediated Inhibition of Oxidative Stress Is Regulated through Nrf2

**Figure 8:**
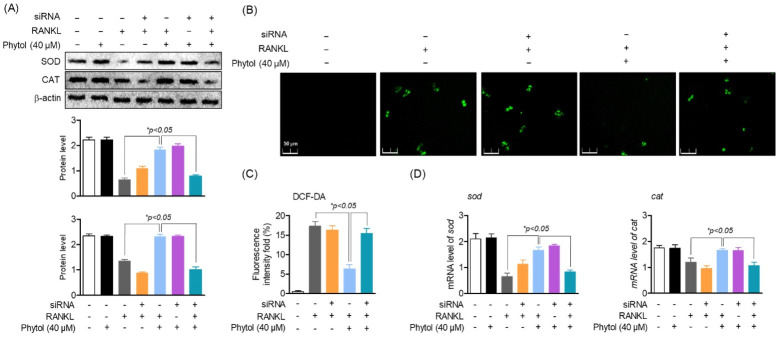
Inhibitory effect of phytol on oxidative stress is regulated through Nrf2. (**A**) In both receptor activator of nuclear factor kappa−B ligand (RANKL)-treated and untreated group, cells were transfected with or without Nrf2 siRNA, in the presence or absence of phytol, and the protein and RNA level of SOD and CAT were investigated using Western blot analysis and RT−PCR, respectively. (**B**,**C**) ROS production was measured in the presence or absence of 40 µM phytol, siRNA silencing, and RANKL stimulation to detect the effect of phytol on oxidative stress through Nrf2. (**D**) The mRNA expression of *sod* and *cat* were detected by RT-PCR. Data are presented as the mean of three independent experiments (Mean ± SD).

Nrf2, a ubiquitous transcription factor and a master regulator of antioxidants, controls the expression of various genes involved in oxidative stress. Thus, to determine the effect of phytol on oxidative stress via Nrf2, ROS screening was performed and antioxidant enzymes were investigated using siRNA. In the presence of RANKL, both SOD and CAT expression significantly decreased (Figure 8A). Similarly, SOD and CAT expression was lower in the RANKL and siRNA treatment group than that in the non-treatment group. Subsequently, the cells were treated with 40 µM phytol with either RANKL or Nrf2 siRNA to recover these antioxidant enzymes. The data also indicated that phytol restored the antioxidant enzyme expression and mRNA levels in the RANKL-induced model. Thus, these data not only confirm that RANKL regulates oxidative stress but also suggest that phytol could suppress the oxidative stress regulated by Nrf2. To confirm this hypothesis, we investigated ROS production. DCF-DA staining (green) indicated that in RANKL-induced osteoclast cells, Nrf2 silencing increased ROS accumulation, which was slightly decreased after treatment with phytol, while ROS accumulation significantly decreased in phytol-treated RANKL-induced osteoclasts without Nrf2 silencing (Figure 8B,C). In addition, it was confirmed that phytol regulates the genes of antioxidant enzymes *sod* and *cat* by Nrf2 regulation (Figure 8D). These data confirm the inhibitory effect of phytol on RANKL-induced osteoclast regulation through Nrf2.

## 4. Discussion

In this study, we investigated the role of phytol in osteoclast differentiation and its association with ROS generation by regulating Nrf2 in RANKL-induced RAW264.7 cells, as Nrf2 is a transcription factor that modulates ROS homeostasis by regulating the expression of antioxidants [6].

As osteoclast differentiation is triggered by increased intracellular ROS generation, osteoclast lineages are prone to oxidative stress; excess ROS levels cause oxidative stress, leading to oxidative damage [6]. Cells try to prevent the oxidant effect and restore the redox balance when oxidative stress occurs by activation or silencing defensive enzymes, transcription factors, and structural protein-encoded genes, and a major mechanism of the cellular defense system against oxidative stress is the activation of Nrf2 [27,28]. Nrf2 is involved in ROS removal by activating antioxidant genes including CAT, SOD, and HO-1 [7,28]. Nrf2-mediated cellular adaptive response has a dual role in RANKL-induced osteoclastogenesis, including the protective roles against oxidative damage and the inhibitory role of RANKL-stimulated ROS signaling [29]. Western blot analysis showed that phytol increased HO-1 expression, based on the concentration and time of Nrf2 translocation to the nucleus, and suppressed ROS accumulation. Moreover, Nrf2 deletion induces oxidative stress and RANKL-induced osteoclast differentiation [17]. In this study, phytol not only inhibited osteospecific structure, function, and ROS accumulation, but also recovered antioxidant enzymes. Our results confirmed that Nrf2 regulation was involved in the inhibition of osteoclast differentiation in the presence of phytol.

Phytol-mediated RANKL-induced osteoclast differentiation inhibition was demonstrated in this study. Mononuclear TRAP-positive cells fuse to form mature osteoclasts, which are the primary cells for bone resorption [30]. In addition, the formation of multinucleated cells and F-actin denote osteoclast-specific structures and bone resorption, respectively [31,32]. Analysis of the TRAP-positive activity and F-actin area showed that phytol suppressed the formation of multinucleated osteoclasts and F-actin concentration-dependently. Since osteoclast precursor migration plays an important role in osteoclast fusion and differentiation [33], bone migration and bone resorption in RANKL-induced RAW264.7 cells processed with phytol was determined to confirm the inhibitory effect of phytol on osteoclast differentiation. RANKL activates several transcription factors including NFATc1 and c-Fos. c-Fos regulation activates NFATc1, a master regulator of osteoclastogenesis [34]. RANKL-induced c-Fos expression plays an important role in osteoclast differentiation initiation. RANKL induces osteoclast precursor differentiation into osteoclasts and ameliorates osteoclast apoptosis via NFATc1 [35]. In this study, the data showed that expression of osteospecific marker genes and transcription factors, including NFATc1 and c-Fos, was inhibited after phytol treatment.

## 5. Conclusions

In conclusion, this study revealed that osteoclast differentiation and function, and the underlying mechanism were suppressed in RANKL-induced RAW 264.7 cells under the effect of phytol, isolated from *S. rebaudiana* leaves. Additionally, Nrf2 silencing data indicated that phytol inhibited osteoclast differentiation through Nrf2 regulation. Our data also indicated that phytol is involved in osteoclast differentiation by suppressing ROS generation and recovering the expression of antioxidant enzymes via Nrf2 regulation. Therefore, phytol is a potential natural product for treating osteoclasts and bone diseases.

## Figures and Tables

**Table 1:** Primer sequences for real-time quantitative PCR analysis.

Target Gene	Sequence (5′→3′)	
*dcstamp*	Forward	TTTGCCGCTGTGGACTATCTGC
	Reverse	GCAGAATCATGGACGACTCCTTG
*acp5*	Forward	CGTCTCTGCACAGATTGCAT
	Reverse	GAGTTGCCACACAGCATCAC
*atp6d0v2*	Forward	TGTGTCCCATTCTTGAGTTTGAGG
	Reverse	AGG GTCTCCCTGTCTTCTTTGCTT
*ctsk*	Forward	TGTATAACGCCACGGCAAA
	Reverse	GGTTCACATTATCACGGTCACA
*sod*	Forward	AACCAGTTGTGTTGTCAGGAC
	Reverse	CCACCATGTTTCTTAGAGTGAGG
*cat*	Forward	AGCGACCAGATGAAGCAGTG
	Reverse	TCCGCTCTCTGTCAAAGTGTG
*gapdh*	Forward	ACAGTCCATGCCATCACTGCC
	Reverse	GCCTGCTTCACCACCTTCTTG

## Data Availability

Not applicable.

## References

[1] Weivoda M.M., Chew C.K., Monroe D.G., Farr J.N., Atkinson E.J., Geske J.R., Eckhardt B., Thicke B., Ruan M., Tweed A.J. (2020). Identification of osteoclast-osteoblast coupling factors in humans reveals links between bone and energy metabolism. Nat. Commun..

[2] Li D., Liu J., Guo B., Liang C., Dang L., Lu C., He X., Cheung H.Y., Xu L., Lu C. (2016). Osteoclast-derived exosomal miR-214-3p inhibits osteoblastic bone formation. Nat. Commun..

[3] Wang Y., Deng P., Liu Y., Wu Y., Chen Y., Guo Y., Zhang S., Zheng X., Zhou L., Liu W. (2020). Alpha-ketoglutarate ameliorates age-related osteoporosis via regulating histone methylations. Nat. Commun..

[4] Wilson C. (2014). Oxidative stress and osteoporosis. Nat. Rev. Endocrinol..

[5] Wells P.G., McCallum P.G., Chen C.S., Henderson J.T., Lee C.J.J., Perstin J., Preston T.J., Wiley M.J., Wong A.W. (2009). Oxidative Stress in Developmental Origins of Disease: Teratogenesis, Neurodevelopmental Deficits, and Cancer. Toxicol. Sci..

[6] Xue P., Hu X., Chang E., Wang L., Chen M., Wu T.H., Lee D.J., Foster B.L., Tseng H.C., Ko C.C. (2021). Deficiency of optineurin enhances osteoclast differentiation by attenuating the NRF2-mediated antioxidant response. Exp. Mol. Med..

[7] Kanzaki H., Shinohara F., Kajiya M., Kodama T. (2014). The Keap1/Nrf2 protein axis plays a role in osteoclast differentiation by regulating intracellular reactive oxygen species signaling. J. Biol. Chem..

[8] Chen X., Zhu X., Wei A., Chen F., Gao Q., Lu K., Jiang Q., Cao W. (2021). Nrf2 epigenetic derepression induced by running exercise protects against osteoporosis. Bone Res..

[9] Kim J.H., Singhal V., Biswal S., Thimmulappa R.K., DiGirolamo D.J. (2014). Nrf2 is required for normal postnatal bone acquisition in mice. Bone Res..

[10] Thanas C., Ziros P.G., Chartoumpekis D.V., Renaud C.O., Sykiotis G.P. (2020). The Keap1/Nrf2 Signaling Pathway in the Thyroid—2020 Update. Antioxidants.

[11] Lu S.H., Chen T.H., Chou T.C. (2015). Magnolol Inhibits RANKL-induced osteoclast differentiation of raw 264.7 macrophages through heme oxygenase-1-dependent inhibition of NFATc1 expression. J. Nat. Prod..

[12] Chen K., Qiu P., Yuan Y., Zheng L., He J., Wang C., Guo Q., Kenny J., Liu Q., Zhao J. (2019). Pseurotin A Inhibits Osteoclastogenesis and Prevents Ovariectomized-Induced Bone Loss by Suppressing Reactive Oxygen Species. Theranostics.

[13] Ando Y., Nakazawa H., Miura D., Otake M., Umetsu M. (2021). Enzymatic ligation of an antibody and arginine 9 peptide for efficient and cell-specific siRNA delivery. Sci. Rep..

[14] Sun Y.X., Li L., Corry K.A., Zhang P., Yang Y., Himes E., Mihuti C.L., Nelson C., Dai G., Li J. (2015). Deletion of Nrf2 reduces skeletal mechanical properties and decreases load-driven bone formation. Bone.

[15] Ibáñez L., Ferrándiz M.L., Brines R., Guede D., Cuadrado A., Alcaraz M.J. (2014). Effects of Nrf2 deficiency on bone microarchitecture in an experimental model of osteoporosis. Oxidative Med. Cell. Longev..

[16] Liu D., Genetos D.C., Shao Y., Geist D.J., Li J., Ke H.Z., Turner C.H., Duncan R.L. (2008). Activation of extracellular-signal regulated kinase (ERK1/2) by fluid shear is Ca(2+)- and ATP-dependent in MC3T3-E1 osteoblasts. Bone.

[17] Hyeon S., Lee H., Yang Y., Jeong W. (2013). Nrf2 deficiency induces oxidative stress and promotes RANKL-induced osteoclast differentiation. Free Radic. Biol. Med..

[18] Lee S., Ryoo R., Choi J.H., Kim J.H., Kim S.H., Kim K.H. (2020). Trichothecene and tremulane sesquiterpenes from a hallucinogenic mushroom *Gymnopilus junonius* and their cytotoxicity. Arch. Pharm. Res..

[19] Lee S.R., Kang H., Yoo M.J., Yu J.S., Lee S., Yi S.A., Beemelmanns C., Lee J., Kim K.H. (2020). Anti-adipogenic pregnane steroid from a *Hydractinia*-associated fungus, *Cladosporium sphaerospermum* SW67. Nat. Prod. Sci..

[20] Lee S., Yu J.S., Phung H.M., Lee J.G., Kim K.H., Kang K.S. (2020). Potential Anti-Skin Aging Effect of (-)-Catechin Isolated from the Root Bark of *Ulmus davidiana* var. *japonica* in Tumor Necrosis Factor-α-Stimulated Normal Human Dermal Fibroblasts. Antioxidants.

[21] Lee K.H., Kim J.K., Yu J.S., Jeong S.Y., Choi J.H., Kim J.C., Ko Y.J., Kim S.H., Kim K.H. (2021). Ginkwanghols A and B, osteogenic coumaric acid-aliphatic alcohol hybrids from the leaves of *Ginkgo biloba*. Arch. Pharm. Res..

[22] Balakrishnan A., Arunambiga S., Praveena A. (2020). Stevia as a Natural Sweetener: A Review. Cardiovasc. Hematol. Agents Med. Chem..

[23] Islam M.T., Ali E.S., Uddin S.J., Shaw S., Islam M.A., Ahmed M.I., Chandra Shill M., Karmakar U.K., Yarla N.S., Khan I.N. (2018). Phytol: A review of biomedical activities. Food Chem. Toxicol..

[24] Coleman R., Body J.J., Aapro M., Hadji P., Herrstedt J. (2014). Bone health in cancer patients: ESMO Clinical Practice Guidelines. Ann. Oncol..

[25] Maraka S.A., Kennel K. (2015). Bisphosphonates for the Prevention and Treatment of Osteoporosis. BMJ.

[26] Ibrahim M.B., Sowemimo A.A., Venables L., Koorbanally N.A., Awolola G.V., Sofidiya M.O., Odukoya O.A., Koekemoer T., van de Venter M. (2018). Biological evaluation of phytoconstituents from *Markhamia tomentosa* ethanolic leaf extract. S. Afr. J. Bot..

[27] Birben E., Sahiner U.M., Sackesen C., Erzurum S., Kalayci O. (2012). Oxidative stress and antioxidant defense. World Allergy Organ. J..

[28] Alemu T.W., Pandey H.O., Salilew W.D., Gebremedhn S., Neuhof C., Tholen E., Holkerm M., Schellander K., Tesfaye D. (2018). Oxidative and endoplasmic reticulum stress defense mechanisms of bovine granulosa cells exposed to heat stress. Theriogenology.

[29] Xue P., Hu X., Powers J., Nay N., Chang E., Kwon J., Wong S.W., Han L., Wu T.H., Lee D.J. (2019). CDDO-Me, Sulforaphane and tBHQ attenuate the RANKL-induced osteoclast differentiation via activating the NRF2-mediated antioxidant response. Biochem. Biophys. Res. Commun..

[30] Zhen G., Dan Y., Wang R., Dou C., Guo Q., Zarr M., Liu L.N., Chen L., Deng R., Li Y. (2021). An antibody against Siglec-15 promotes bone formation and fracture healing by increasing TRAP^+^ mononuclear cells and PDGF-BB secretion. Bone Res..

[31] Oh E., Lee H.Y., Kim H.J., Park Y.J., Seo J.K., Park J.S., Bae Y.S. (2015). Serum amyloid A inhibits RANKL-induced osteoclast formation. Exp. Mol. Med..

[32] Matsubara T., Kinbara M., Maeda T., Yoshizawa M., Kokabu S., Takano Y.T. (2017). Regulation of osteoclast differentiation and actin ring formation by the cytolinker protein plectin. Biochem. Biophys. Res. Commun..

[33] Liang Z., Xue Y., Wang T., Xie Q., Lin J., Wang Y. (2020). Curcumin inhibits the migration of osteoclast precursors and osteoclastogenesis by repressing CCL3 production. BMC Complement. Med. Ther..

[34] AlQranei M.S., Aljohani H., Majumdar S., Senbanjo L.T., Chellaiah M.A. (2020). C-phycocyanin attenuates RANKL-induced osteoclastogenesis and bone resorption in vitro through inhibiting ROS levels, NFATc1 and NF-κB activation. Sci. Rep..

[35] Kang I.S., Kim C. (2016). NADPH oxidase gp91phox contributes to RANKL-induced osteoclast differentiation by upregulating NFATc1. Sci. Rep..

